# Supplementary material for: Prevalence and Preferred Niche of Small Eukaryotes with Mixotrophic Potentials in the Global Ocean
Source: Microorganisms. 2024 Apr 8;12(4):750. doi: 10.3390/microorganisms12040750 (PMC11051772; doi:10.3390/microorganisms12040750)
Supplement: Supplementary file 1 [file microorganisms-12-00750-s001.zip › microorganisms-2900183-supplementary.pdf]

Supplementary material for the manuscript, 'Prevalence and Preferred Niche of Small Eukaryotes with Mixotrophic Potentials in the Global Ocean', accepted for publication in the *Microorganisms* journal by Dong et al.

Supplementary Table S1. Species and cellular 18S rRNA gene copy numbers used in this study to retrieve gene-cell abundance correction factors for small eukaryotes.

Taxa	Group	Reference	Method	Biovolume (μm^3)	Gene Copies per Cell
Ostreococcus tauri	other eukaryotes	Gong et al. 2019	bioinformatics	0.5	3.4
Trebouxia sp.		Gong et al. 2019	bioinformatics	1767.1	78.1
Bathycoccus prasinos		Zhu et al. 2005	qPCR	3.8	5.2
Emiliania huxleyi		Zhu et al. 2005	qPCR	64.3	2.5
Mesopedinella arctica		Zhu et al. 2005	qPCR	252.8	7.4
Micromonas pusilla		Zhu et al. 2005	qPCR	4.0	3.0
Nannochloropsis salina		Zhu et al. 2005	qPCR	4.0	1.0
Ostreococcus tauri		Zhu et al. 2005	qPCR	0.3	2.5
Pelagomonas calceolata		Zhu et al. 2005	qPCR	7.7	1.3
Rhodomonas salina		Zhu et al. 2005	qPCR	217.0	28.8
Tetraselmis sp.		Zhu et al. 2005	qPCR	208.7	7.6
Chrysochromulina sp.		Li et al., 2022	qPCR	20.6	2.0
Chrysochromulina sp.		Li et al., 2022	qPCR	41.6	2.0
ChrysoH		Li et al., 2022	qPCR	5.6	1.0
DictyX		Li et al., 2022	qPCR	78.0	4.5
Florenciella Parvula		Li et al., 2022	qPCR	26.5	1.2
Hap2		Li et al., 2022	qPCR	47.7	3.0
Rhizochromulina		Li et al., 2022	qPCR	14.1	1.2
Triparma		Li et al., 2022	qPCR	8.2	0.8
Florenciella sp.		Li et al., 2022	qPCR	31.1	2.0
Symbiodinium kawagutii	dinoflagellate	Gong et al. 2019	bioinformatics	288.7	160.5
Symbiodinium minutum		Gong et al. 2019	bioinformatics	220.9	116.0
Amphidinium carterae		Zhu et al. 2005	qPCR	432.0	83.7
Prorocentrum nux		Zhu et al. 2005	qPCR	79.9	22.1
Extubocellulus spinifer	diatom	Godhe et al. 2008	qPCR	150.0	19.0
Phaeodactylum triconutum		Gong et al. 2019	bioinformatics	101.6	4.2
Thalassiosira oceaniaca		Gong et al. 2019	bioinformatics	179.5	17.2
Nitzschia closterium		Zhu et al. 2005	qPCR	640.0	28.0
Thalassiosira weissflogii		Zhu et al. 2005	qPCR	471.0	14.8

Supplementary Table S2. Stepwise regression analysis revealed significant variables contributing to the prevalence of mixotrophs in both surface and chlorophyll maximal layer (SUR and CML) samples, and surface (SUR) samples alone. Significance of P values and coefficient for 8 selected predictors were given in each row.

SUR and CML			SUR		
**Predictor**[†]	**P**[‡]	**Coefficient**	**Predictor**[†]	**P**[‡]	**Coefficient**
PAR	**	0.1	CML	***	0.15
NO_3^- and NO_2^-	***	-0.08	*Prochlorococcus*	**	-0.82
Density	***	0.80	NO_3^-&NO_2^-	***	-0.15
PIC	**	207.5	PIC	*	218.3
CML	***	0.10	PO_4^{3-}	*	0.28
Hbac	**	1.25	Density	*	0.78
Prochlorococcus	*	-0.53	PAR	**	0.12
/	/	/	NPP	.	0.08

[†]Abbreviations: PAR, photosynthetically available radiation; PIC, particulate inorganic carbon; Hbac, heterotrophic bacteria; NPP, net primary production

[‡] Significant codes: '***' p<0.001, '**' p<0.01, '*' p<0.05, '.' p<0.1

Supplementary figures
Figure S1. Redundancy analysis for environmental impact on the distribution of trophic groups in surface oceans, derived from 18S rRNA gene corrected cell abundances. Only 17 *Tara Oceans* lineages passed Escoufier selectin (0.9 threshold) were shown, and adjusted R^2 of the analysis is of 0.54. Environmental variables selected were denoted in cyan-blue arrows and text. Abbreviations: PAR, photosynthetically active radiations; CML, chlorophyll maximum layer; MLD, mixed layer depth; POC, particulate organic carbon; PIC, particulate inorganic carbon; Pro, *Prochlorococcus*.
Figure S2. Linear regression between $TI_{A/H}$ (all in cell abundances) and resource variables retrieved from the SUR (104 samples) and SUR and CML dataset (165 samples). Different significance levels were marked with different number of red stars (one star if $0.001<p<0.05$ and two stars if $p<0.001$), and shaded bands are pointwise 95% confidence interval on the fitted values (the line). Abbreviations: PAR, photosynthetically active radiations; N: inorganic nutrients of nitrate (NO_3^-) and nitrite (NO_2^-); Hbac: heterotrophic bacteria; CML, chlorophyll maximal layer.
Figure S3. Linear regression between TI_g (all in cell abundances) and single environmental variables that have shown significant correlations, retrieved from all 165 SUR and CML samples. Different significance levels were marked with different number of red stars (one star if $0.001<p<0.05$ and two stars if $p<0.001$), and shaded bands are pointwise 95% confidence interval on the fitted values (the line). Abbreviations: CML, chlorophyll maximal layer; Chl *a*, total chlorophyll *a*, Pro, *Prochlorococcus*.

Figure S1

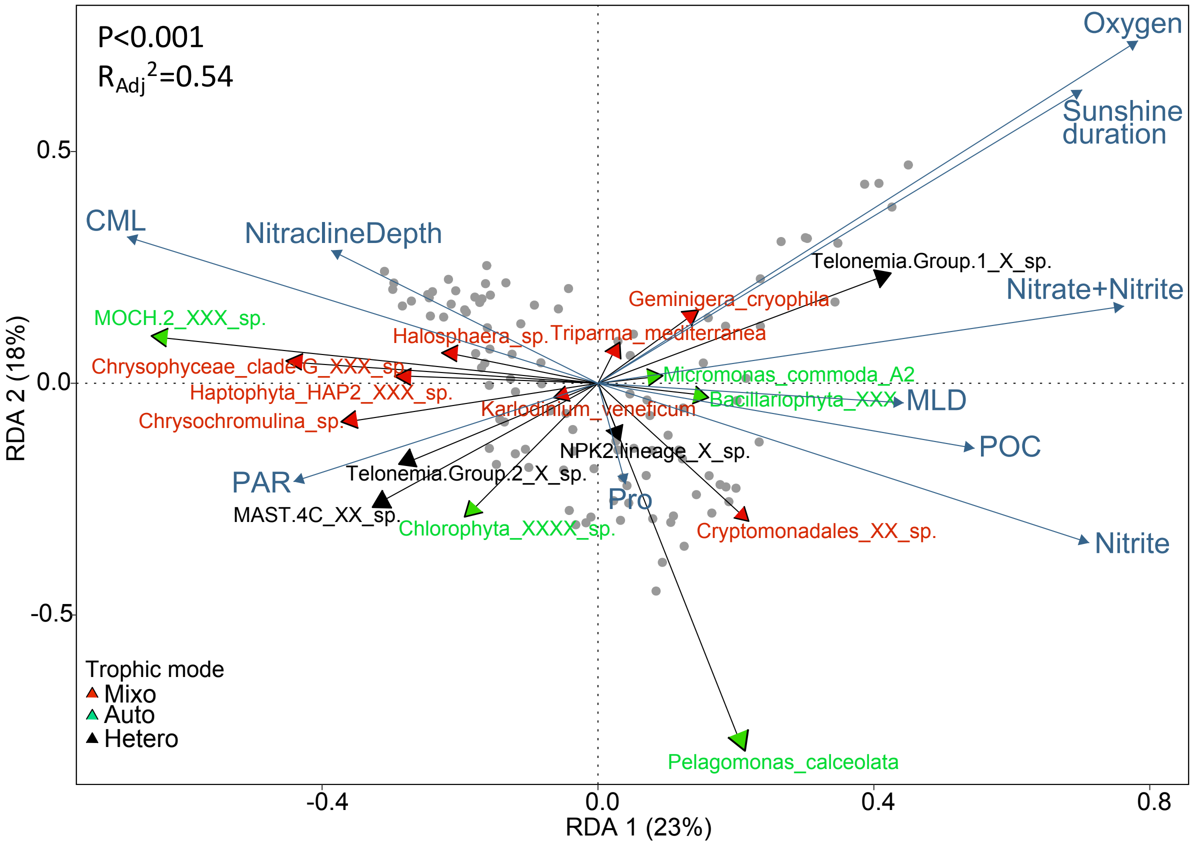
P<0.001
R_Adj^2=0.54
Oxygen
Sunshine duration
CML
NitraclineDepth
Telonemia.Group.1_X_sp.
Nitrate+Nitrite
Geminigera_cryophila
MOCH.2_XXX_sp.
Halosphaera_sp.
Triparma_mediterranea
Chrysophyceae_clade G_XXX_sp.
Micromonas_commoda_A2
Haptophyta_HAP2_XXX_sp.
Bacillariophyta_XXX
MLD
Karlodinium_veneficum
Chrysochromulina_sp.
POC
NPK2.lineage_X_sp.
PAR
Telonemia.Group.2_X_sp.
Pro
MAST.4C_XX_sp.
Chlorophyta_XXXX_sp.
Cryptomonadales_XX_sp.
Nitrite
Trophic mode
Mixo
Auto
Hetero
Pelagomonas_calceolata
0.5
0.0
-0.5
-0.4
0.0
0.4
0.8
RDA 1 (23%)
RDA 2 (18%)

Figure S2

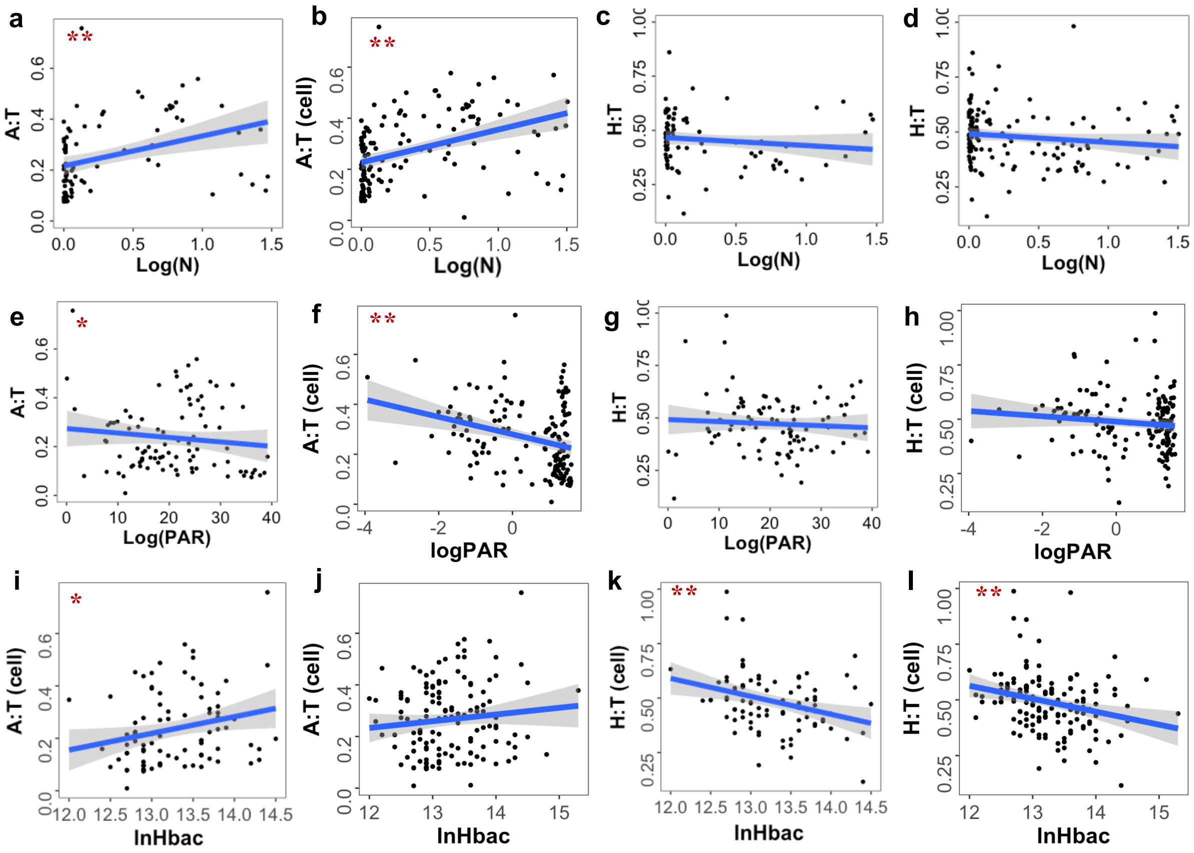
a
b
c
d
e
f
g
h
i
j
k
l
A:T
A:T (cell)
H:T
H:T (cell)
Log(N)
Log(PAR)
logPAR
lnHbac

Figure S3

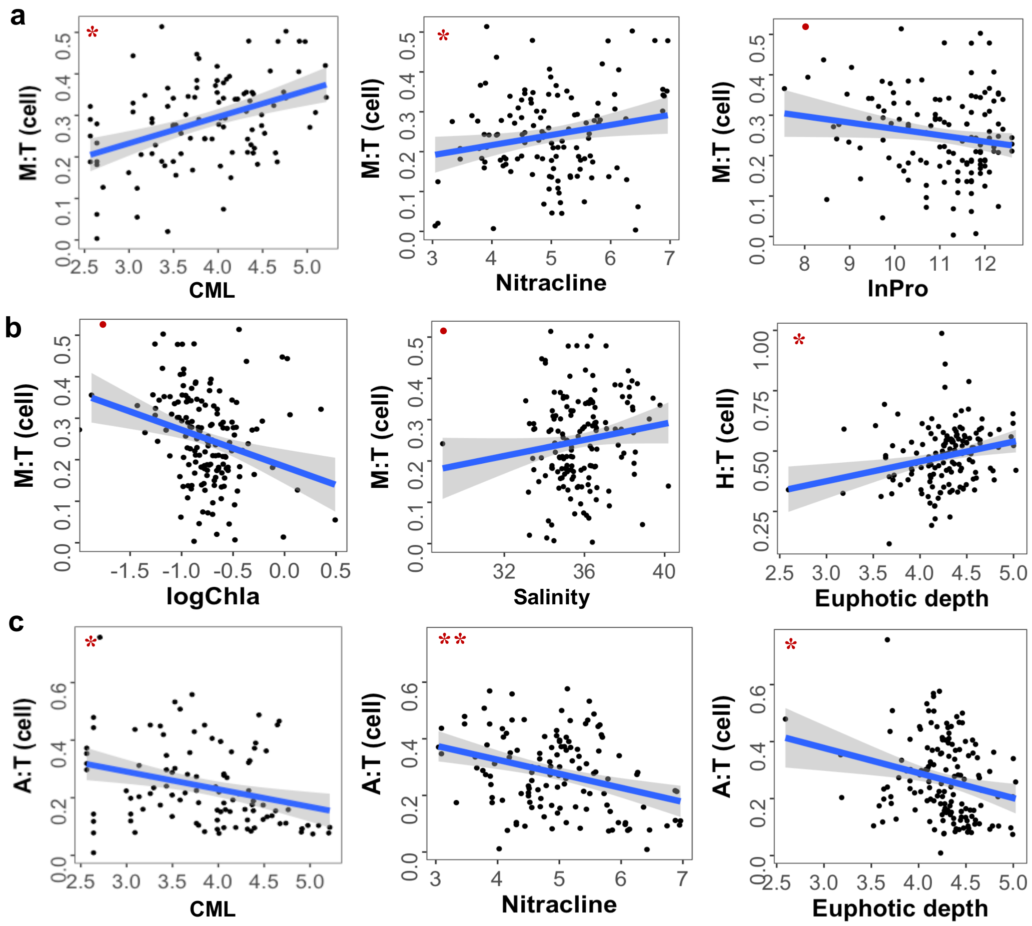
a
M:T (cell)
CML
M:T (cell)
Nitracline
M:T (cell)
InPro
b
M:T (cell)
logChla
M:T (cell)
Salinity
H:T (cell)
Euphotic depth
c
A:T (cell)
CML
A:T (cell)
Nitracline
A:T (cell)
Euphotic depth

References

Godhe, A., Asplund, M. E., Härnström, K., Saravanan, V., Tyagi, A. and Karunasagar, I. (2008). Quantification of diatom and dinoflagellate biomasses in coastal marine seawater samples by real-time PCR. Appl. Environ. Microbiol. 74: 7174–7182. doi:10.1128/AEM.01298-08

Gong, W.D. and Marchetti, A. (2019). Estimation of 18S Gene Copy Number in Marine Eukaryotic Plankton Using a Next-Generation Sequencing Approach. Front. Mar. Sci., Sec. Mar. Mol. Bio. Ecol. doi: 10.3389/fmars.2019.00219

Li, Q., Edwards, K. F., Schvarcz, C. R. and Steward., G. F. (2022). Broad phylogenetic and functional diversity among mixotrophic consumers of Prochlorococcus. The ISME Journal 16(6): 1557–1569. doi:10.1038/s41396–022–01204–z

Zhu, F., Massana, R., Not, F., Marie, D. and Vaulot, D. (2005). Mapping of picoeucaryotes in marine ecosystems with quantitative PCR of the18S rRNA gene. FEMS in Microbial Ecology 52(1): 79–92. doi:10.1016/j.femsec.2004.10.006
